# Prevalence and correlates of restrictive interventions in an Irish child and adolescent psychiatric unit: a 4-year retrospective study

**DOI:** 10.1007/s11845-023-03316-7

**Published:** 2023-02-23

**Authors:** Maeve Haran, David Killeen, Mike Healy, Peadar Brophy, Aoife Donohue, Imelda Whyte, Brendan Doody

**Affiliations:** 1 Department of Psychiatry, School of Medicine, University College Dublin, Dublin, Ireland; 2 Children’s Health Ireland at Crumlin, Dublin, D12N512 Ireland; 3 Dublin North City and County CAMHS - Ballymun CAMHS, Ballymun Civic Centre, Dublin, Ireland; 4 Linn Dara Inpatient Unit, Cherry Orchard Hospital, Ballyfermot Rd, Cherry Orchard, Dublin, Ireland; 5 Department of Psychiatry, Trinity College Dublin, Dublin, Ireland

**Keywords:** Hospital, Mental health, Physical restraint, Seclusion, Youth

## Abstract

**Background:**

There has been a global effort to reduce the use of restrictive interventions (RIs) in healthcare settings. In order to reduce unnecessary RIs, it is essential to understand their use in mental health settings. To date, there have been few studies examining the use of RIs in child and adolescent mental health settings, with no such studies in Ireland.

**Aims:**

The purpose of this study is to examine the prevalence and frequency of physical restraints and seclusion and to identify any associated demographic and clinical characteristics.

**Methods:**

This is a 4-year retrospective study of the use of seclusion and physical restraint in an Irish child and adolescent psychiatric inpatient unit from 2018 to 2021. Computer-based data collection sheets and patient records were retrospectively reviewed. Eating disorder and non-eating disorder samples were analysed.

**Results:**

Of 499 hospital admissions from 2018 to 2021, 6% (*n* = 29) had at least one episode of seclusion and 18% (*n* = 88) had at least one episode of physical restraint. Age, gender and ethnicity were not significantly associated with rates of RI. Unemployment, prior hospitalization, involuntary legal status and longer length of stay were significantly associated with higher rates of RIs in the non-eating disorder group. Involuntary legal status was associated with higher rates of physical restraint in the eating disorder group. Patients with a diagnosis of eating disorder and psychosis had the highest prevalence of physical restraints and seclusions respectively.

**Conclusions:**

Identifying youth who are at greater risk of requiring RIs may allow early and targeted intervention and prevention.

**Supplementary Information:**

The online version contains supplementary material available at 10.1007/s11845-023-03316-7.

## Introduction

There has been an international effort to reduce the use of restrictive interventions (RIs) in healthcare settings [1–4]. RIs—defined as “planned or reactive acts that restrict an individual’s movement, liberty or freedom to act independently” [2] — include physical restraint (PR) and seclusion, and are used in child and adolescent psychiatric units worldwide. Ireland’s Mental Health Commission (MHC) defined PR as the “use of physical force for the purpose of preventing the free movement of a resident’s body” and seclusion as “placing or leaving a person alone in a room with the exit door locked or held in such a way as to prevent the person from leaving”[5]. Violence and aggression in mental health services most commonly occur in inpatient settings and can have significant impacts on the patient, other patients in the hospital, and staff [6]. When non-restrictive measures have failed, the use of RIs may be necessary to protect service users and staff from harm [2]. RIs may also be used as a last resort to facilitate the treatment of eating disorders (EDs) [7, 8]. However, the use of RIs can lead to psychological trauma and physical injuries [9–11]. Therefore, its use poses an ethical conflict between a child’s right to autonomy liberty and integrity, and a child’s right to protection from physical violence, and also to their right to life, survival and development [12].

The United Nations’ (UN) Convention on the Rights of Persons with Disabilities (CRPD) and the World Health Organization (WHO) have suggested strategies to reduce the use of RIs [13, 14]. By ratifying the CRPD in 2018, Ireland made a commitment to its principles and values, which includes addressing the use of RIs in mental health settings [15]. In Ireland in 2020, there was a total of 5830 episodes of PR (83.8 per 100,000) and seclusion (34.3 per 100,000) recorded nationally across all approved centres, including both child and adolescent and adult inpatient units. PR was used in 72.7% and seclusion in 41% of all inpatient units. Our study site had the highest number of PRs (601) recorded in 2021 which Ireland’s MHC explained was due to a small number of residents being restrained on a frequent basis [16].

In 2014, the MHC presented a framework with strategies aiming to sustain a reduction in RIs in approved centres. They listed eight key strategies, which include setting up working groups, training staff in de-escalation techniques, involving patient and families in care planning, debriefing after an episode and ensuring compliance with regulations set out by the MHC. One of the eight strategies that the MHC also emphasised was the use of data and they urged mental health services to analyse the use of RIs in their own institutions [4].

We know that in order to reduce unnecessary RIs, it is essential to understand their use in mental health settings. To date, there have been few studies examining the use of RIs in child and adolescent mental health settings with no such studies in Ireland.

A systematic review by Nielson et al. highlighted that certain patient characteristics including gender, age, diagnosis and length of stay (LOS) were significantly associated with rates of PR of children and adolescents in psychiatric units [17]. The only European studies included in this study originated in Finland and Norway. The purpose of this quantitative study is to contribute to the literature published on RIs. Our aim is to examine the prevalence of PR and seclusion and to assess associated demographic and clinical characteristics in a child and adolescent psychiatric inpatient unit in Ireland. We hope that our results will help inform policy development around reducing the use of RI.

## Methods

### Design

This is a 4-year retrospective study of the use of seclusion and PR in an Irish child and adolescent psychiatric inpatient unit from the 1st of January 2018 to the 31st of December 2021. Nursing managers in Linn Dara Approved Centre log every application of PR, mechanical restraint and seclusion on a computer-based system. Data on every patient admitted to the inpatient unit is also tracked on a computer-based system.

A discharge diagnosis for each admission is determined, using ICD-10 (International Classification of Diseases 10th Revision), by the consultant child and adolescent psychiatrist managing the patient and these diagnoses are recorded in the computer-based system. Diagnoses were compressed into 8 groups to aid analysis; mood or anxiety disorders (including bipolar affective disorder), psychotic disorders, EDs, emotional and behavioural disorders, personality disorders, mental and behavioural disorder due to substance misuse, disorders of psychological development and other disorders.

The lead investigator used a data collection sheet that was developed for this study. For every admission, data was extracted from these computer-based systems on both frequency and durations of RIs (PRs, episodes of seclusion and mechanical restraints) along with clinical data detailing primary psychiatric discharge diagnosis, legal status on admission, order of admission, LOS and demographic information. This was done in tandem with file reviews to retrieve any missing data. For those who were not yet discharged on 31.12.21, their LOS was calculated from date of admission until 31.12.21. Previous hospitalizations included admissions in general hospital settings prior to transfer to the inpatient unit.

### Participants

The sample comprised all hospital admissions (*n* = 499) between 1st of January 2018 and 31st of December 2021 in Linn Dara Approved Centre in Dublin, Ireland. Linn Dara Approved Centre is a 24-bedded inpatient psychiatric unit with 2 high-dependency beds and 8 specialist ED beds, which is run and funded by the Health Service Executive. This approved centre treats children and adolescents, under the age of 18 years old, primarily from the eastern seaboard and midlands area including Dublin city, which comprises a total population of over 2 million.

### Data analyses

Prevalence rates were calculated by dividing the number of admissions during which a person was secluded or restrained by the total number of admissions (*n* = 499). Statistical analyses were run on SPSS. Chi-squared tests were used to compare the proportion of patients secluded and restrained in each category of the independent variables: age, gender, ethnicity, legal status on admission, occupation, order of admission, LOS, year of admission and discharge diagnosis. These associations were separately analysed in the ED sample and the non-ED sample.

## Results

### Demographic and clinical characteristics

The mean age of the sample was 15.26 years (SD = 1.559). Almost two-thirds of the total sample admitted was female with a higher proportion of females (93%) in the ED group. The majority were white Irish and students. Over 70% of the total sample were first admissions and over 90% were voluntary patients on admission. The mean LOS was 47.72 days (SD = 72.895). The majority of total admissions had a LOS less than 30 days; however, the ED sample mostly had 30–120 day admissions (Table 1).

**Table 1:** Demographic and clinical characteristics of the total, non-eating disorder, and eating disorder samples

**Socio-demographic characteristic**	**Total sample** ***n*** **(%)**	**Non-ED sample** ***n*** **(%)**	**ED sample** ***n*** **(%)**
Total sample	499 (100)	427 (100)	72 (100)
Age group (years)			
5–11	12 (2.4)	9 (2.1)	3 (4.2)
12–15	234 (46.9)	192 (45.0)	42 (58.3)
16–17	253 (50.7)	226 (52.9)	27 (37.5)
Gender			
Female	317 (63.5)	250 (58.5)	67 (93.1)
Male	182 (36.5)	177 (41.5)	5 (6.9)
Ethnicity			
White Irish	411 (82.4)	347 (81.3)	64 (88.9)
Occupation			
Student	470 (94.2)	398 (93.2)	72 (100)
In training/employed	4 (0.8)	4 (0.9)	0
Unemployed	25 (5.0)	25 (5.9)	0
**Clinical characteristic**			
Order of admission			
First admission	364 (72.9)	306 (71.7)	58 (80.6)
Previous hospitalisation	135 (27.1)	121 (28.3)	14 (19.4)
Legal status			
Voluntary	459 (92.0)	393 (92.0)	66 (91.7)
Involuntary	40 (8.0)	34 (8.0)	6 (8.3)
LOS			
< 30 days	295 (59.1)	287 (67.2)	8 (11.1)
30–120 days	162 (32.5)	125 (29.3)	37 (51.4)
> 120 days	42 (8.4)	15 (3.5)	27 (37.5)

### Prevalence and duration of RIs

Of 499 admissions, 18% (*n* = 88) had at least one episode of PR and 6% of admissions (*n* = 29) had at least one episode of seclusion. This amounted to 1868 episodes of PR and 186 episodes of seclusion between 2018 and 2021. Of the 29 admissions who were secluded, all but one (*n* = 28) also had an episode of PR (Table S1). Comparing non-ED and ED samples, the prevalence of seclusion was 6% in both and the rate of PR was higher in the ED sample (38%) compared to the non-ED sample (14%). Almost one-third (*n* = 603) of episodes of PR occurred in the ED sample. In the total sample, the mean duration of PR was 8.65 min and seclusion was 8.3 h. The mean duration of PR was lower, 8.3 min, in the non-ED sample.

### Distribution of RIs

Five admissions accounted for 67% episodes of seclusions (*n* = 125). Of these, 60% (*n* = 3) were 16 or 17 years olds and 60% (*n* = 3) were male. Eighty percent (*n* = 4) were voluntary on admission and 80% (*n* = 4) had previous hospitalizations. There were 40% (*n* = 2) with a LOS under 30 days, 20% (*n* = 1) between 30 and 120 days and 40% (*n* = 2) over 120 days. Forty percent (*n* = 2) had a primary diagnosis of disorders of psychological development; the remainder had a psychotic disorder, a behavioural and emotional disorder and a mental and behavioural disorder secondary to substance misuse.

Five admissions accounted for 57% (*n* = 1063) of PRs. Of these 60% (*n* = 3) were 16–17 years old and 80% (*n* = 4) were female. Eighty percent (*n* = 4) were voluntary on admission, 60% (*n* = 3) were first admissions and all five had a LOS over 120 days. Forty percent (*n* = 2) had a diagnosis of an ED with the remainder having diagnoses of personality disorder, mood or anxiety disorder and a behavioural and emotional disorder.

### Frequency of RIs

Of those secluded, 31% (*n* = 9) had only one episode throughout their admission, 38% (*n* = 11) had 2–4 episodes, 10% (*n* = 3) had 5–9 episodes and 21% (*n* = 6) required 10 or more episodes. Of the 88 admissions requiring a PR, over one-third (*n* = 32, 36%) only had one episode, 23% (*n* = 20) had 2–4 episodes, 17% (*n* = 14) had 5–9 episodes and 24% (*n* = 21) had 10 or more episodes of PR (Table S5).

### Influence of age, gender and ethnicity on RIs

There were no significant associations between age, gender or ethnicity, and the prevalence rates or frequency of RIs. In the non-ED sample, males had a nonsignificant higher prevalence of both PRs and seclusion. In comparison, females had a nonsignificant higher prevalence of PR and seclusion than males in the ED group. Amongst the total group, females were more likely to be restrained more frequently than males (*p* = 0.024) and there was no statistical difference in the frequency of seclusions between genders (Tables S1 and S2).

### Influence of occupation on RIs

In the non-ED group, there was a significant association between occupation status and seclusion rates (*p* = 0.036). Unemployed youths had the highest rate of seclusion (20%, *n* = 5) compared to students (5%, *n* = 20) or those in training or employed (0.0%, *n* = 0). There was no significant difference in rates of PR or frequencies of RIs between the groups (Table 2, S1 S2).

**Table 2:** Demographic and clinical correlates of seclusion and physical restraint in non-eating disorder sample and eating disorder sample

**Variable**	**Seclusion in non-ED sample (*n*** **= 427)** ***n*** **(%)**	***p***	**Seclusion in ED sample** **(*n*** **= 72)** ***n*** **(%)**	***p***	**PR in non-ED sample** **(*n*** **= 472)** ***n*** **(%)**	***p***	**PR in ED sample (*n*** **= 72)** ***n*** **(%)**	***p***
**Age:**								
5–11	0 (0)	0.658^a^	1 (33.3)	0.666^a^	2 (22.2)	0.444^a^	2 (66.7)	0.368^a^
12–15	9 (4.7)		3 (7.1)		24 (12.5)		17 (40.5)	
16–18	16 (7.1)		0		35 (15.5)		8 (29.6)	
**Gender:**								
Male	14 (7.9)	0.128	0	0.745^a^	27 (15.3)	0.630	1 (20.0)	0.644^a^
Female	11 (4.4)		4 (6.0)		34 (13.6)		26 (38.8)	
**Ethnicity:**								
White Irish	18 (5.2)	0.286^a^	4 (6.3)	0.618^a^	47 (13.5)	0.362	26 (40.6)	0.244^a^
All other	7 (8.8)		0		14 (17.5)		1 (12.5)	
**Occupation**								
Student	20 (5.0)	0.036^a^*	4 (5.6)	n/a	54 (13.6)	0.141	27 (37.5)	n/a
Training/employed	0 (0)		n/a		0 (0)		n/a	
Unemployed	5 (20.0)		n/a		7 (28.0)		n/a	
**Order of admission**								
First admission	13 (4.2)	0.037*	3 (5.2)	1.0	36 (11.8)	0.018*	20 (34.5)	0.282
Previous hospitalisation	12 (9.9)		1 (7.1)		25 (20.7)		7 (50.0)	
**Legal status on admission**								
Voluntary	20 (5.1)	0.039^a^*	4 (6.1)	1.0	50 (12.7)	0.004^a^*	22 (33.3)	0.025^a*^
Involuntary	5 (14.7)		0		11 (32.4)		5 (83.3)	
**LOS**								
< 30 days	15 (5.2)	0.002^a^*	0	0.429	25 (8.7)	< 0.001^a^*	3 (37.5)	0.351^a^
30–120 days	5 (4.0)		1 (2.7)		26 (20.8)		11 (29.7)	
> 120 days	5 (33.3)		3 (11.1)		10 (66.7)		13 (48.1)	
**Year of admission**								
2018	4 (6.8)	0.827^a^	0	0.220^a^	7 (11.9)	0.749	1 (10.0)	0.044^a*^
2019	5 (4.2)		3 (15.8)		20 (14.8)		10 (52.6)	
2020	8 (6.6)		0		18 (12.6)		9 (52.9)	
2021	8 (6.3)		1 (3.8)		16 (14.3)		7 (26.9)	

*p* values were calculated using Pearson chi-squared tests

^*^p value < 0.05

^a^If the expected counts were less than 5, then Fisher’s exact test of independence was used to calculate *p* value

### Influence of year of admission on RIs

There was no significant difference in the prevalence or frequency of seclusion or PRs between 2018 and 2021 in the non-ED group (Table 2, S1, S2). In the ED sample, there were more PRs in 2019 and 2020 (53% in both year) compared to 2018 (10%) and 2021 (27%), *p* = 0.044 (Table 2).

### Influence of legal status on admission

Involuntary status admissions were associated with more PRs in both the non-ED group (32% *n* = 11, *p* = 0.004) and the ED group (83%, *n* = 5, *p* = 0.025) and with more seclusions (15%, *n* = 5, *p* = 0.039) in the non-ED group (Table 2). There were no significant differences in the frequency of RIs based on the legal status of admission (Tables S3 and S4).

### Influence of previous hospitalisation on RIs

Those with previous hospitalizations had a significantly higher rate of PR (10%, *n* = 12, *p* = 0.037) than first ever admissions (4%, *n* = 13) in the non-ED group only. They also had a significantly higher rate of seclusion (21%, *n* = 25, *p* = 0.018) than first admissions (12%, *n* = 36) (Table 2). There were no significant differences in the ED sample nor in frequency of RIs between the groups (Tables S3 and S4).

### Influence of LOS on RIs

In the non-ED sample, LOS was significantly associated with prevalence of RIs, with rates of PR and seclusion increasing with longer lengths of stay (Table 2). Of those requiring a PR, longer lengths of stays were associated with more frequent restraints (*p* ≤ 0.001); however, the frequency of seclusions was not statistically associated with LOS (Tables S3 and S4).

### Influence of discharge diagnosis on RIs

**Fig. 1:**
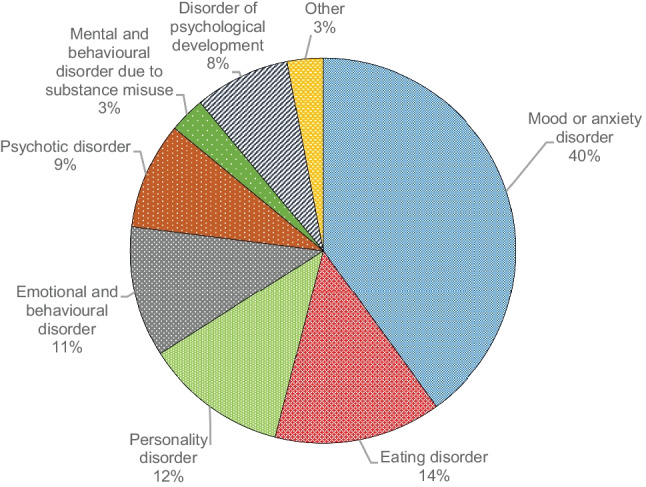
Primary discharge diagnosis in the total sample

**Fig. 2:**
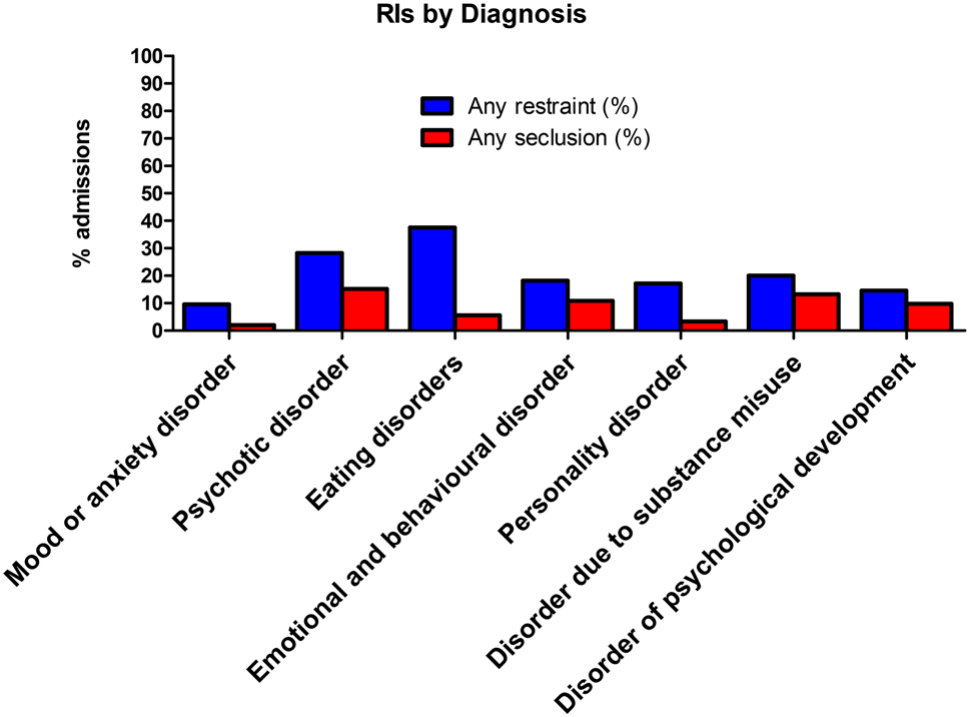
Prevalence of PR and seclusion by discharge diagnosis

The most common primary discharge diagnosis was a mood or anxiety disorder (40%, *n* = 197) (Fig. 1). Discharge diagnosis was significantly associated with prevalence of PR and seclusion. ED diagnoses had the highest proportion of PRs with 37.5% (*n* = 27) requiring a restraint and psychotic disorders had the highest proportion of seclusion (15.2%, *n* = 7) (Fig. 2).

## Discussion

This is the first study that has analysed the prevalence and associated demographic and clinical risk factors for PR and seclusion in an inpatient child and adolescent mental health service in an Irish context.

In our study, across 4 years, the prevalence of PR was 18% in the total sample, 14% in the non-ED sample and 38% in the ED sample. Nielson et al.’s systematic review cited higher figures of 27–44% of patients physically restrained in inpatient child and adolescent mental health services from five studies across the USA, Europe and Australia [17]. In our group, 6% of total admissions required at least one episode of seclusion. De Hert et al.’s systematic review of seven studies reported a higher baseline rate of seclusion with a weighted mean of 26% ranging from 8.5 to 61% of patients [18]. Although our rates of PR and seclusion are lower than those demonstrated in these systematic reviews, it is difficult to interpret this difference due to the heterogeneity of these studies. Both systematic reviews have included older studies dating as far back as 2002 [19], and given the global focus on reducing RIs over the last 20 years, we would expect lower rates in our study.

We found a higher proportion of PRs (1868 episodes across 4 years) compared to seclusions (186 episodes), which was in contrast to other studies demonstrating higher rates of seclusion compared to restraint in inpatient child and adolescent mental health units [20–23]. In line with previous research [24–27], our study found the distribution of RIs was skewed by a small number of inpatients with frequent RIs, with five admission accounting for 57% of PRs and five admissions accounting for 67% of seclusions. The majority of these had LOSs over 120 days and had been previously hospitalized.

The mean duration of PR in our study was 8.65 min and 8.30 min in the non-ED group, which is higher than Ireland’s national average of 6 min across all approved centres, both child and adolescent and adult inpatient units in 2020 [16]. The mean duration of seclusion (8.3 h) was lower than a national average of 14 h and 12 min across all approved centres except forensic settings in 2020 [16]. Due to multiple variables between sites and lack of data collected about indications for episodes, we cannot draw inferences from these comparisons.

Similar to other studies, we found that prior hospitalisation [26, 28], involuntary legal status on admission [28] and a longer LOS [19, 24, 26–30] were associated with significantly higher prevalence rates of both PR and seclusion in the non-ED sample. Unemployment status was also significantly associated with higher prevalence of seclusion in the non-ED sample. This is in line with research, mostly in adult populations, highlighting that unemployment is a risk factor for coercive interventions in inpatient care [31–33]. Rates of PR within the ED sample increased in 2019 and 2020. It is notable that our study site, Linn Dara Approve Centre, introduced nasogastric feeding as an intervention for ED in 2019 and this may account for the spike in rates during this development period.

In our cohort, admissions with a primary diagnosis of eating disorder had the highest prevalence of PR and amounted to almost one-third (*n* = 603) of the total episodes PRs over the 4 years. Similarly, a Danish based study, of all admissions to psychiatric and somatic wards, reported the majority of PRs in under 18 year olds was in patients with EDs [8]. Our rates may reflect the high proportion of specialist eating disorder beds (8 of 24 beds) in our study site. Patients with a diagnosis of an eating disorder are admitted to these beds to receive treatment which may include nasogastric feeding. If a patient is refusing this treatment, PR may be required, as a last resort, to facilitate this potentially lifesaving treatment. These children are subject to orders under the Mental Health Act (MHA) authorising the use of PR to facilitate the administration this treatment. Eight percent of our ED sample were admitted under the MHA, and these were significantly more likely to receive a PR than their voluntary counterparts. A 2014 systematic review of inpatients, across all ages, with severe anorexia nervosa, reported that 13–44% required involuntary treatment such as nasogastric feeding or mental health committal [34]. Compared to 38% of EDs requiring PR in our study, a recent study carried out at a specialist Norwegian ED facility found, a slightly lower, 32% of child and adolescent inpatients required restraint during their admission [35].

Those with a diagnosis of psychotic disorder and mental and behavioural disorder due to substance misuse had the highest rates of seclusion and the next highest rates of PR after EDs. We have insufficient data to conclude reasons for these findings; however, possible hypotheses include the associated increased risks of violent behaviour and involuntary treatments amongst these groups. According to Van Dorn et al., a diagnosis of a substance use disorder increases the risk of violence compared to the general public [35]. Similarly, a large meta-analysis indicated that psychosis is associated with an increased risk of violence [36]. In addition, there is evidence that schizophrenia is a risk factor for the administration of medication under restraint [37]. A Canadian cohort study of first episode psychosis (*n* = 17,725), reported that 26% of patients required an involuntary admission within the course of their illness [38]. Consistent with our study, psychosis has been identified as a risk factor for restraints in a previous study across four American child and adolescent inpatient units [26] and psychosis has been the most predictive diagnostic indicator for the use of seclusion in adult patients [39]. Conversely, some studies in child and adolescent units have cited attention deficit and disruptive disorders [40] and developmental disorders [41] as most likely to be secluded and restrained. While some studies have found no association between diagnosis and restraints or seclusion [24, 27, 42]. One study found that psychotic, eating and externalising disorders predicted the use of RIs in their sample but when adjusted for Children’s Global Assessment Scale (CGAS) scores they were no longer significant predictors, suggesting that psychosocial functioning rather than diagnosis is a more important risk factor [28].

National and international initiatives to reduce the use of seclusion and restraint focus on preventative management strategies. Identifying modifiable risk factors that may predispose children and adolescents to PR and seclusion is a crucial step to reach these goals. This study suggests that children at risk of RIs may be identified on admission which could aid in individual care planning, undertaking structured risk assessments and then managing staffing levels appropriately.

## Limitations

This was a single-site study, which limits its generalisability. These results may not generalise to other inpatient units that do not admit acutely, have differing admission criteria or those that do not administer nasogastric gastric feeding. It was a retrospective review of both clinical records and data collected on computer-based systems. Data may not have been inputted uniformly by staff which may have caused information bias.

Due to the very small number of patients receiving mechanical restraint, the data was not analysed nor presented as patient confidentiality would be at risk. Data was not collected on the indications for RIs, severity of illness nor environmental factors such as staff or institution characteristics which may be important influencing factors and thus limits interpretation of results. We did not collect qualitative data from patients or clinicians which would be helpful to understand the impact of RIs on these groups. Subsample sizes may have been too small to demonstrate any statistically significant association between ethnicity, age and rate of RIs.

## Conclusions

This study provides data on prevalence rates, and the demographic and clinical factors, that are associated with PR and seclusion in an Irish child and adolescent mental health service inpatient unit. Our findings carry practical considerations for identifying youth who are at greater risk of requiring RIs so that appropriate preventative measures may be considered, and resources allocated as early as possible during inpatient admission. Further research is required to explore the characteristics associated with the use of RIs in the ED cohort. It would be important, also, for future research to analyse the impact of RIs on both patients and staff and gain insight into perceptions about RI reduction strategies.

### Supplementary Information

Below is the link to the electronic supplementary material.

Supplementary file1 (DOCX 34 KB)

## Data Availability

The authors declare that all data supporting the findings of this study are available within the article and it's supplementary information file.
