# Supplementary material for: Prevalence and correlates of restrictive interventions in an Irish child and adolescent psychiatric unit: a 4-year retrospective study
Source: Ir J Med Sci. 2023 Feb 23;192(6):2929–36. doi: 10.1007/s11845-023-03316-7 (PMC10692034; doi:10.1007/s11845-023-03316-7)
Supplement: Supplementary file 1 — Supplementary file1 (DOCX 34 KB) [file 11845_2023_3316_MOESM1_ESM.docx]

**Prevalence and correlates of restrictive interventions in an Irish child and adolescent psychiatric hospital: a 4-year retrospective study**

*Maeve Haran^1^, David Killeen^2^, Mike Healy^3^, Peadar Brophy^3^, Aoife Donohue^3^, Imelda Whyte^3^, Brendan Doody^3, 4.^

1. Department of Psychiatry, School of Medicine, University College Dublin, Dublin, Ireland.

2. Dublin North City and County CAMHS - Ballymun CAMHS, Ballymun Civic Centre, Dublin, Ireland.

3. Linn Dara Inpatient Unit, Cherry Orchard Hospital, Ballyfermot Rd, Cherry Orchard, Dublin, Ireland.

4. Department of Psychiatry, Trinity College Dublin, Ireland

*Address for Correspondence:

Dr Maeve Haran,

Senior Psychiatry Registrar,

Children's Health Ireland at Crumlin

Dublin D12N512, Ireland.

[maeve.haran@ucd.ie](mailto:maeve.haran@ucd.ie)

**Supplemental Information**

| **Table S1** | **Frequency of physical restraints episodes by demographic characteristics** |
| --- | --- |
| **Table S2** | **Frequency of seclusion episodes by demographic characteristics** |
| **Table S3** | **Frequency of physical restraints episodes by clinical characteristics** |
| **Table S4** | **Frequency of seclusion episodes by clinical characteristics** |
| **Table S5** | **Number of physical restraints and seclusions by diagnosis** |

**Table S1** *Frequency of physical restraints episodes by demographic characteristics*

| Characteristic | Number of physical restraints episodes | | | | | | | Total | Fisher’s exact p value |
| --- | --- | --- | --- | --- | --- | --- | --- | --- | --- |
|  | 1  n (%) | | 2-4  n (%) | 5-9  n (%) | | >9  n (%) | |  |  |
| Total sample | 32 (36) | 20 (23) | | | 15 (17) | | 21 (24) | 88 (100) |  |
| Age |  |  | | |  | |  |  |  |
| 5-11 | 1 (25.0) | 1 (25.0) | | | 1 (25.0) | | 1 (25.0) | 4 | 0.206 |
| 12-15 | 12 (29.3) | 8 (19.5) | | | 11 (26.8) | | 10 (24.4) | 41 |  |
| 16-18 | 19 (44.2) | 11(25.6) | | | 3 (7.0) | | 10 (23.3) | 43 |  |
| Gender |  |  | | |  | |  |  |  |
| Male | 15 (53.6) | 5 (17.9) | | | 6 (21.4) | | 2 (7.1) | 28 | 0.024* |
| Female | 17 (28.3) | 15 (25.0) | | | 9(15.0) | | 19(31.7) | 60 |  |
| Ethnicity |  |  | | |  | |  |  |  |
| White Irish | 25 (34.2) | 17 (23.3) | | | 12 (16.4) | | 19 (26.0) | 73 | 0.708 |
| All other ethnicities | 7 (46.7) | 3 (20.0) | | | 3 (20.0) | | 2 (13.3) | 15 |  |
| Place of birth |  |  | | |  | |  |  |  |
| Ireland | 29 (35.8) | 19 (23.5) | | | 13 (16.0) | | 20 (24.7) | 81 | 0.755 |
| Great Britain | 1 (100) | 0 | | | 0 | | 0 | 1 |  |
| Rest of Europe | 1 (50) | 1 (50) | | | 0 | | 0 | 2 |  |
| Africa | 0 | 0 | | | 1 (50) | | 1 (50) | 2 |  |
| Asia | 1 (50) | 0 | | | 1 (50) | | 0 | 2 |  |
| South America |  |  | | |  | |  |  |  |
| Occupation |  |  | | |  | |  |  |  |
| Student | 29 (35.8) | 16 (19.8) | | | 15 (18.5) | | 21 (25.9) | 81 | 0.080 |
| Unemployed | 3 (42.9) | 4 (57.1) | | | 0 | | 0 | 7 |  |
| Year of admission |  |  | | |  | |  |  |  |
| 2018 | 2 (25.0) | 1 (12.5) | | | 1 (12.5) | | 4 (50.0) | 8 | 0.249 |
| 2019 | 9 (30.0) | 7 (23.3) | | | 3 (10.0) | | 11 (36.7) | 30 |  |
| 2020 | 12 (44.4) | 7 (25.9) | | | 6 (22.2) | | 2 (7.4) | 27 |  |
| 2021 | 9 (39.1) | 5 (21.7) | | | 5 (21.7) | | 4 (17.4) | 23 |  |

*N = number of admissions*

*P values were calculated using Pearson Chi squared tests. If the expected counts were less than 5, then Fisher's exact test of independence were used to calculate P value.*

**Table S2** *Frequency of seclusion episodes by demographic characteristics*

| Characteristic | Number of seclusion episodes | | | | | | | Total n | Fisher’s exact p value |
| --- | --- | --- | --- | --- | --- | --- | --- | --- | --- |
|  | 1  n (%) | | 2-4  n (%) | | 5-9  n (%) | | >9  n (%) |  |  |
| Total sample | 9 (31) | | 11 (38) | | 3 (10) | | 6 (21) | 29 (100) |  |
| Age |  |  | |  | |  | |  |  |
| 5-11 | 1 (100) | 0 | | 0 | | 0 | | 1 | 0.777 |
| 12-15 | 4 (33.3) | 4 (33.3) | | 2 (16.7) | | 2 (16.7) | | 12 |  |
| 16-18 | 4 (25.0) | 7 (43.8) | | 1 (6.3) | | 4 (25.0) | | 16 |  |
| Gender |  |  | |  | |  | |  |  |
| Male | 3 (21.4) | 6 (42.9) | | 2 (14.3) | | 3(21.4) | | 14 | 0.698 |
| Female | 6 (40.0) | 5 (33.3) | | 1 (6.7) | | 3 (20.0) | | 15 |  |
| Ethnicity |  |  | |  | |  | |  |  |
| White Irish | 8 (36.4) | 7 (31.8) | | 1 (4.5) | | 6 (27.3) | | 22 | 0.101 |
| All other ethnicities | 1 (14.3) | 4 (57.1) | | 2 (28.6) | | 0 | | 7 |  |
| Place of birth |  |  | |  | |  | |  |  |
| Ireland | 9 (32.1) | 11 (39.3) | | 2 (7.1) | | 6 (21.4) | | 28 |  |
| Great Britain | 0 | 0 | | 0 | | 0 | | 0 |  |
| Rest of Europe | 0 | 0 | | 0 | | 0 | | 0 |  |
| Africa | 0 | 0 | | 1 (100) | | 0 | | 1 |  |
| Asia | 0 | 0 | | 0 | | 0 | | 0 |  |
| South America | 0 | 0 | | 0 | | 0 | | 0 |  |
| Occupation |  |  | |  | |  | |  |  |
| Student | 6 (25.0) | 10 (41.7) | | 3 (12.5) | | 5 (20.8) | | 24 | 0.477 |
| Unemployed | 3(60.0) | 1(20.0) | | 0 | | 1(20.0) | | 5 |  |
| Year of admission |  |  | |  | |  | |  |  |
| 2018 | 1 (25) | 1 (25) | | 0 | | 2 (50) | | 4 | 0.080 |
| 2019 | 4 (50) | 1 (12.5) | | 2 (25) | | 1 (12.5) | | 8 |  |
| 2020 | 2 (25.0) | 2 (25.0) | | 1 (12.5) | | 3 (37.5) | | 8 |  |
| 2021 | 2 (22.2) | 7 (77.8) | | 0 | | 0 | | 9 |  |

*N = number of admissions*

*P values were calculated using Pearson Chi squared tests. If the expected counts were less than 5, then Fisher's exact test of independence were used to calculate P value.*

**Table S3** *Frequency of physical restraints episodes by clinical characteristics*

| Characteristic | Number of physical restraints episodes | | | | Total | Fisher’s exact p value |
| --- | --- | --- | --- | --- | --- | --- |
|  | 1  n (%) | 2-4  n (%) | 5-9  n (%) | >9  n (%) |  |  |
| Total sample | 32 (36) | 20 (23) | 15 (17) | 21 (24) | 88 (100) |  |
| Order of admission |  |  |  |  |  |  |
| First admission | 26 (46.4) | 10 (17.9) | 9 (16.1) | 11 (19.6) | 56 | 0.069 |
| Previous hospitalisation | 6 (18.8) | 10 (31.3) | 6(18.8) | 10 (31.3) | 32 |  |
| Legal status on admission |  |  |  |  |  |  |
| Voluntary | 26 (36.1) | 18 (25.0) | 11 (15.3 | 17 (23.6) | 72 | 0.667 |
| Involuntary | 6 (37.5) | 2 (12.5) | 4 (25.0) | 4 (25.0) | 16 |  |
| Length of stay |  |  |  |  |  |  |
| <30 days | 16 (57.1) | 7 (25.0) | 2 (7.1) | 3 (10.7) | 28 | <0.001* |
| 30-120 days | 12 (32.4) | 10 (27.0) | 11 (29.7) | 4 (10.8) | 37 |  |
| >120 days | 4 (17.4) | 3 (13.0) | 2 (8.7) | 14 (60.9) | 23 |  |
| Primary diagnosis |  |  |  |  |  |  |
| Mood or anxiety disorder | 9 (47.4) | 3 (15.8) | 4 (21.1) | 3 (15.8) | 19 | 0.568 |
| Psychotic disorder | 4 (30.8) | 5 (38.5) | 2 (15.4) | 2 (15.4) | 13 |  |
| Eating disorder | 11 (40.7) | 5 (18.5) | 3 (11.1) | 8 (29.6) | 27 |  |
| Emotional and behavioural disorder | 4 (40.0) | 3 (30.0) | 2 (20.0) | 1 (10.0) | 10 |  |
| Personality disorder | 3 (30.0) | 1 (10.0) | 2 (20.0) | 4 (40.0) | 10 |  |
| Mental and behavioural disorder due to substance misuse | 1 (33.3) | 2(66.7) | 0 | 0 | 3 |  |
| Disorder of psychological development | 0 | 1 (16.7) | 2 (33.3) | 3 (50.0) | 6 |  |
| Other | 0 | 0 | 0 | 0 | 0 |  |

*N = number of admissions*

*P values were calculated using Pearson Chi squared tests. If the expected counts were less than 5, then Fisher's exact test of independence were used to calculate P value.*

**Table S4** *Frequency of seclusion episodes by clinical characteristics*

| Characteristic | Number of seclusion episodes | | | | | | | | Fisher’s exact p value |
| --- | --- | --- | --- | --- | --- | --- | --- | --- | --- |
|  | 1 | | 2-4 | | 5-9 | | >9 | Total |  |
|  |  |  |  |  |  |  |  |  |  |
|  | n (%) | n (%) | | n (%) | | n (%) | |  |  |
| Total sample | 9 (31) | 11 (38) | | 3 (10) | | 6 (21) | | 29 (100) |  |
| Order of admission |  |  | |  | |  | |  |  |
| First admission | 7 (43.8) | 6 (37.5) | | 1 (6.3) | | 2 (12.5) | | 16 | 0.350 |
| Previous hospitalisation | 2 (15.4) | 5 (38.5) | | 2 (15.4) | | 4 (30.8) | | 13 |  |
| Legal status on admission |  |  | |  | |  | |  |  |
| Voluntary | 9 (37.5) | 7 (29.2) | | 3 (12.5) | | 5 (20.8) | | 24 | 0.202 |
| Involuntary | 0 | 4 (80) | | 0 | | 1 (20) | | 5 |  |
| Length of stay |  |  | |  | |  | |  |  |
| <30 days | 5 (33.3) | 6 (40.0) | | 2 (13.3) | | 2 (13.3) | | 15 | 0.747 |
| 30-120 days | 1 (16.7) | 3 (50.0) | | 1 (16.7) | | 1 (16.7) | | 6 |  |
| >120 days | 3 (37.5) | 2 (25.0) | | 0 | | 3 (37.5) | | 8 |  |
| Primary diagnosis |  |  | |  | |  | |  |  |
| Mood or anxiety disorder | 2 (50) | 2 (50) | | 0 | | 0 | | 4 | 0.789 |
| Psychotic disorder | 1 (14.3) | 2 (28.6) | | 2 (28.6) | | 2 (28.6) | | 7 |  |
| Eating disorder | 3 (75) | 1 (25) | | 0 | | 0 | | 4 |  |
| Emotional and behavioural disorder | 2 (33.3) | 2 (33.3) | | 1 (16.7) | | 1 (16.7) | | 6 |  |
| Personality disorder | 1 (50) | 1 (50) | | 0 | | 0 | | 2 |  |
| Mental and behavioural disorder due to substance misuse | 0 | 1 (50) | | 0 | | 1 (50) | | 2 |  |
| Disorder of psychological development | 0 | 2 (50) | | 0 | | 2 (50) | | 4 |  |
| Other | 0 | 0 | | 0 | | 0 | | 0 |  |

*N = number of admissions*

*P values were calculated using Pearson Chi squared tests. If the expected counts were less than 5, then Fisher's exact test of independence were used to calculate P value.*

**Table S5** *Number of physical restraints and seclusions by diagnosis*

| Diagnosis | Number of physical restraints | Number of seclusions |
| --- | --- | --- |
| Mood and anxiety disorder including BPAD | 412 | 6 |
| Psychotic disorder | 81 | 38 |
| Eating disorder | 603 | 5 |
| Behavioural and emotional disorders | 278 | 50 |
| PD | 292 | 4 |
| Substance | 6 | 15 |
| Disorders of psychological development | 196 | 68 |
| Other | 0 | 0 |
